# Comparative Analysis of Clinical and Metabolic Profiles in Ischemic Versus Hemorrhagic Stroke Among Adults Presenting to a Tertiary Care Hospital

**DOI:** 10.7759/cureus.90245

**Published:** 2025-08-16

**Authors:** Tayyaba Arooj Mufti, Hafiz Zunair Iqbal, Ammar Nawazish, Amara Sajjad, Shiv Priya, Fatima Shaukat, Ayesha Farooq, Muhammad Zaryab Haider, Muhammad Ayoob Memon, Muhammad Irfan Jamil, Adeel Ahmed, Iqra Naeem

**Affiliations:** 1 Cardiology/Medicine, Akhtar Saeed Medical and Dental College, Lahore, PAK; 2 Internal Medicine, Akhtar Saeed Medical and Dental College, Lahore, PAK; 3 Internal Medicine, Avicenna Hospital Lahore, Lahore, PAK; 4 Medicine, Asaaf Hospital Lahore, Lahore, PAK; 5 Critical Care Medicine, St George's University Hospital, London, GBR; 6 Medicine, Lahore General Hospital, Lahore, PAK; 7 Medicine, Ameeruddin Medical College Lahore, Lahore, PAK; 8 Internal Medicine, Sir Gangaram Hospital Lahore, Lahore, PAK; 9 Cardiology, Rawalpindi Institute of Cardiology, Rawalpindi, PAK; 10 Acute Internal Medicine, Blackpool Teaching Hospitals NHS Foundation Trust, Blackpool, GBR; 11 Internal Medicine, Jinnah Sindh Medical University, Karachi, PAK; 12 Nephrology, Lahore General Hospital, Lahore, PAK; 13 Anesthesiology and Critical Care, Lahore General Hospital, Lahore, PAK; 14 Psychiatry and Behavioral Sciences, Lahore General Hospital, Lahore, PAK

**Keywords:** hemorrhagic cva, ischemic cva, mortality, prevalence studies, risk factor stroke, stroke

## Abstract

Aim and background: Stroke remains a major cause of mortality and morbidity, with substantial heterogeneity in risk factors and outcomes by subtype. This study aimed to compare the prevalence and clinical profiles of ischemic and hemorrhagic strokes and to evaluate the distribution of demographic, neurological, clinical, and metabolic variables among adults presenting within 24 hours of symptom onset.

Materials and methods: A cross-sectional observational study was conducted at a tertiary care hospital in Lahore from June 2023 to July 2024. A total of 800 adults diagnosed with ischemic or hemorrhagic stroke were enrolled using non-probability consecutive sampling. Demographic, clinical, and laboratory parameters, including body mass index (BMI), Glasgow Coma Scale (GCS), blood pressure, lipid profile, glycated hemoglobin (HbA1c), and renal function, were recorded within 24 hours of admission.

Results: In this study of 800 patients, ischemic stroke was identified in 565 (70.6%) and hemorrhagic stroke in 235 (29.4%). Patients aged 50 years or younger had significantly lower odds of ischemic stroke compared to those above 50 years (OR = 0.515, p < 0.001). No significant association was observed between gender and stroke type. High socioeconomic status was associated with a greater proportion of hemorrhagic stroke (χ² = 19.69, p < 0.001). Ever smokers had higher odds of ischemic stroke (OR = 1.405, 95% CI: 1.027-1.921, p = 0.033). Hypertension (HTN) was significantly more frequent in hemorrhagic stroke (OR = 1.453, 95% CI: 1.055-2.000, p = 0.022), while diabetes mellitus (DM) and dyslipidemia showed no significant association with stroke type. Chronic kidney disease was not significantly associated with hemorrhagic stroke (OR = 1.678, 95% CI: 0.996-2.826, p = 0.050). Presentation within 4.5 hours of symptom onset was significantly associated with hemorrhagic stroke (OR = 2.993, 95% CI: 2.034-4.405, p < 0.001). Systolic blood pressure (SBP) and diastolic blood pressure (DBP) were significantly higher in hemorrhagic stroke (SBP mean difference (MD) = -31.30, 95% CI: -36.02 to -26.58, p < 0.001; DBP MD = -10.01, 95% CI: -14.74 to -5.28, p < 0.001). Ischemic stroke patients had higher total cholesterol, low-density lipoprotein cholesterol (LDL-C), and triglycerides, while hemorrhagic stroke patients had higher high-density lipoprotein cholesterol (HDL-C) and blood urea. In-hospital mortality occurred in 74 (13.1%) of the ischemic stroke group and 57 (24.3%) of the hemorrhagic stroke group, with significantly higher mortality associated with hemorrhagic stroke (OR = 2.17, 95% CI: 1.47-3.20, p < 0.001). Mortality was also associated with advanced age, lower Glasgow Coma Scale score, higher systolic blood pressure, and elevated cholesterol and triglyceride levels.

Conclusion: This study highlights distinct demographic, clinical, and metabolic profiles between ischemic and hemorrhagic stroke. Hypertension, high socioeconomic status, and early hospital presentation were associated with hemorrhagic stroke, while ischemic stroke was more prevalent among older patients and smokers. In-hospital mortality was significantly higher in hemorrhagic stroke and was linked to advanced age, reduced Glasgow Coma Scale score, and adverse metabolic parameters.

## Introduction

Stroke, also referred to as cerebrovascular accident (CVA), remains one of the foremost causes of mortality and long-term disability across the globe, exerting a substantial burden on healthcare resources. Clinically, strokes are classified into two major categories: ischemic stroke, caused by arterial occlusion leading to cerebral infarction, and hemorrhagic stroke, which results from spontaneous rupture of cerebral vessels, manifesting as intracerebral or subarachnoid hemorrhage [1,2]. Globally, ischemic stroke constitutes approximately 76% of all stroke cases, with hemorrhagic subtypes, including intracerebral and subarachnoid hemorrhages, accounting for the remainder. In 2019, there were an estimated 101.5 million individuals living with stroke worldwide - 77.2 million with ischemic stroke and 20.7 million with intracerebral hemorrhage [3]. This pattern is reflected in Pakistan, where hospital-based data indicate that approximately 71% of strokes among older adults are of the ischemic type [4]. Despite slight reductions in age-standardized stroke incidence rates, the absolute prevalence continues to escalate, driven largely by increasing age and the rising prevalence of modifiable risk factors such as hypertension, dyslipidemia, and physical inactivity [5-7].

Although ischemic and hemorrhagic strokes share several risk factors, their clinical and metabolic profiles often diverge. Ischemic strokes are more commonly associated with atherosclerotic and cardiometabolic abnormalities, including elevated low-density lipoprotein cholesterol, total cholesterol, and triglyceride levels [6,7]. Conversely, hemorrhagic strokes are more frequently linked to chronic hypertension, alcohol use, and underlying vascular fragility, particularly in younger individuals [8]. Previous studies from a range of regional and international settings have consistently demonstrated these distinctions, with ischemic stroke patients typically exhibiting higher lipid parameters and hemorrhagic stroke patients presenting with more severe hypertension and greater neurological impairment at the time of admission, as evidenced by higher scores on established clinical severity scales and increased early mortality [5,6,9]. Additionally, poorer functional outcomes and increased short-term mortality have been documented more often following hemorrhagic stroke, despite its lower overall incidence [10].

With the continued rise in diabetes (DM), hypertension (HTN), sedentary lifestyles, and other risk factors, cardiovascular diseases such as stroke are increasing in prevalence [5]. Therefore, it is necessary to provide updated data on the prevalence of stroke subtypes in the current population. In addition, differences in risk factors, clinical presentation, and metabolic profiles have a significant impact on the type of stroke and on in-hospital mortality. This study provides contemporary data by examining the distribution of key demographic, clinical, and metabolic characteristics in both ischemic and hemorrhagic stroke, and evaluates the relationship of these variables with clinical outcomes. The results contribute important insights for optimizing risk assessment and management in acute stroke.

## Materials and methods

Study design, duration and setting

A cross-sectional observational study was conducted in the Department of Medicine at Lahore General Hospital, Lahore. The study period extended from June 2023 to July 2024. Ethical approval was obtained from the Institutional Review Board (IRB # 106/06/2023), and written informed consent was acquired from all participants or their legally authorized representatives prior to enrollment. Non-probability consecutive sampling was used to enroll eligible patients presenting to the emergency and inpatient departments.

Inclusion and exclusion criteria 

A total of 800 adult patients aged 18 years or above, presenting within 24 hours of the onset of acute neurological symptoms, were included. Diagnosis of ischemic or hemorrhagic stroke was established using clinical assessment and neuroimaging [11]. Ischemic stroke was defined as infarction in a vascular territory confirmed on repeat non-contrast computed tomography (CT) brain after 24 hours, following initial exclusion of hemorrhage. Hemorrhagic stroke was confirmed by identification of hyperdense blood on non-contrast CT in parenchymal, ventricular, or subarachnoid spaces. Exclusion criteria comprised transient ischemic attack without radiological evidence, stroke mimics (such as hypoglycemia, trauma, postictal paralysis, brain tumors, migraine with aura, and conversion disorder), incomplete clinical or laboratory data, inadequate imaging, pregnancy, and those leaving against medical advice. 

Data collection procedure

Baseline demographic characteristics recorded at admission included age, gender, residence (urban/rural), socioeconomic status (categorized as low: <25,000 PKR, middle: 25,000-75,000 PKR, high: >75,000 PKR monthly income), and body mass index (BMI, kg/m²), calculated from measured weight and height. Smoking status was categorized as ever smoker (current/former) or non-smoker. Clinical history was evaluated for comorbidities, including hypertension (systolic blood pressure ≥140 mmHg, diastolic blood pressure ≥90 mmHg, or current antihypertensive use), DM (glycated hemoglobin (HbA1c) ≥6.5%), dyslipidemia (total cholesterol ≥200 mg/dL, low-density lipoprotein cholesterol (LDL-C) ≥130 mg/dL, high-density lipoprotein cholesterol (HDL-C) <40 mg/dL for males and <50 mg/dL for females, triglycerides ≥150 mg/dL, or lipid-lowering therapy) and chronic kidney disease (estimated glomerular filtration rate <60 mL/min/1.73 m² for at least three months or documented diagnosis). Clinical characteristics assessed included stroke type, anatomical site of lesion on imaging, level of consciousness using the Glasgow Coma Scale (GCS), and neurological signs (loss of consciousness, seizures). Blood pressure (systolic and diastolic) was measured within one hour of admission. Laboratory investigations were performed within 24 hours of admission, including total cholesterol, LDL-C, HDL-C, triglycerides, non-HDL cholesterol, fasting and random glucose, HbA1c, serum creatinine, blood urea, uric acid and sodium. All results were recorded in standard units. In-hospital mortality was defined as any death occurring prior to hospital discharge. Data recorded using data collection proforma.

Statistical analysis 

Continuous variables were expressed as mean and standard deviation. Categorical variables were reported as frequencies and percentages. Group comparisons were conducted using the chi-square test for categorical variables and the independent samples t-test for continuous variables. Univariate analysis was performed to assess the association of demographic, clinical, and metabolic variables with in-hospital mortality, presenting odds ratios with 95% confidence intervals. Statistical significance was set at p < 0.05. Data analysis performed through SPSS version 26.0 (IBM Corp., Armonk, NY, USA).

## Results

A total of 800 patients were included, with ischemic stroke identified in 565 (70.6%) and hemorrhagic stroke in 235 (29.4%). The mean age was 63.91 ± 13.40 years, and most patients were older than 50 years. Males comprised nearly two-thirds of the patients. The majority had low or middle socioeconomic status and an urban background. The mean BMI was 26.57 ± 4.88 kg/m², with the majority of the patients in the overweight category. HTN and DM were observed in about one-third and one-fourth of patients, respectively. Most patients arrived at the hospital after 4.5 hours from symptom onset, with a mean arrival time of 8.98 ± 4.80 hours. The basal ganglia was the most frequent lesion site. The mean GCS score was 9.82 ± 2.49, with mean systolic and diastolic blood pressures of 163.19 ± 34.07 mmHg and 101.71 ± 31.36 mmHg, respectively. In-hospital mortality occurred in 131 (16.4%) patients (Table 1).

**Table 1:** Baseline Demographic, Clinical, and Neurological Characteristics of Study Participants (n=800).

Variable	Frequency (%)
Age Group	
≤50 years	174 (21.8%)
>50 years	626 (78.3%)
Gender	
Male	515 (64.4%)
Female	285 (35.6%)
Socioeconomic Status	
Low	364 (45.5%)
Middle	295 (36.9%)
High	141 (17.6%)
Residence	
Rural	341 (42.6%)
Urban	459 (57.4%)
Body Mass Index (kg/m²)	
<18.5 (Underweight)	35 (4.4%)
18.5–24.9 (Normal)	266 (33.3%)
25.0–29.9 (Overweight)	285 (35.6%)
≥30.0 (Obese)	214 (26.8%)
Smoking Status	
Ever smoker	339 (42.4%)
Never smoker	461 (57.6%)
Hypertension	
No	544 (68.0%)
Yes	256 (32.0%)
Diabetes Mellitus	
No	606 (75.8%)
Yes	194 (24.3%)
Dyslipidemia	
No	596 (74.5%)
Yes	204 (25.5%)
Chronic Kidney Disease	
No	735 (91.9%)
Yes	65 (8.1%)
Arrived within 4.5 hours of symptom onset	
No	671 (83.9%)
Yes	129 (16.1%)
Lesion Site	
Cortical (cerebral cortex)	170 (21.3%)
Subcortical (white matter, internal capsule)	178 (22.3%)
Basal ganglia	201 (25.1%)
Thalamus	104 (13.0%)
Brainstem	83 (10.4%)
Cerebellum	33 (4.1%)
Multifocal	31 (3.9%)
Seizures at Presentation	
No	712 (89.0%)
Yes	88 (11.0%)
Stroke Subtype	
Ischemic	565 (70.6%)
Hemorrhagic	235 (29.4%)
In-hospital Mortality	
No	669 (83.6%)
Yes	131 (16.4%)

Age group was significantly associated with stroke subtype, as patients aged ≤50 years were less likely to have ischemic stroke compared to those over 50 years (OR = 0.515, 95% CI: 0.363-0.731, p < 0.001). Gender distribution did not differ significantly between ischemic and hemorrhagic stroke (OR = 1.007, 95% CI: 0.733-1.384, p = 0.964). Ever smokers had higher odds of ischemic stroke than never smokers (OR = 1.405, 95% CI: 1.027-1.921, p = 0.033). Presentation within 4.5 hours was significantly associated with hemorrhagic stroke (OR = 2.993, 95% CI: 2.034-4.405, p < 0.001). Hypertension was more common in hemorrhagic stroke (OR = 1.453, 95% CI: 1.055-2.000, p = 0.022), while dyslipidemia and diabetes mellitus were not significantly associated with stroke subtype (dyslipidemia: OR = 0.798, 95% CI: 0.558-1.142, p = 0.217; diabetes: OR = 0.876, 95% CI: 0.611-1.256, p = 0.470). Seizures at presentation were significantly more common in hemorrhagic stroke (OR = 2.209, 95% CI: 1.408-3.468, p < 0.001). Ischemic stroke patients had higher BMI, longer time to hospital arrival, and better Glasgow Coma Scale scores. They also exhibited significantly higher total cholesterol, low-density lipoprotein, triglycerides, serum uric acid, and sodium levels. In contrast, hemorrhagic stroke patients had significantly elevated systolic and diastolic blood pressures, higher high-density lipoprotein, and increased blood urea levels. HbA1c showed no significant difference (Table 2).

**Table 2:** Association of Demographic and Clinical Variables with Stroke Subtype (n=800). For each variable and subgroup, the table presents the absolute frequency and column-wise percentage of ischemic and hemorrhagic stroke patients, as well as the mean and standard deviation (SD) for continuous variables. The odds ratio (OR) or mean difference (MD) with 95% confidence interval (CI) quantifies the association. The relevant t-test or Pearson’s chi-square (χ²) statistic and p-value are shown, with p < 0.05 considered statistically significant. Abbreviations: BMI, body mass index; GCS, Glasgow Coma Scale; LDL, low-density lipoprotein; HDL, high-density lipoprotein; HbA1c, glycated hemoglobin. Percentages are column-wise for each stroke type.

Variable	Subgroup	Ischemic n (%) (n=565)	Hemorrhagic n (%) (n=235)	OR/MD (95% CI)	t-test/ χ²	p-value
Age (continues) years		65.68 ± 12.58	59.65 ± 14.35	6.03 (4.03 to 8.03)	5.923	< 0.001
Age Group (years)	≤50	103 (18.2%)	71 (30.2%)	0.515 (0.363–0.731)	14.00	< 0.001
	>50	462 (81.8%)	164 (69.8%)			
Gender	Male	364 (64.4%)	151 (64.3%)	1.007 (0.733–1.384)	0.002	0.964
	Female	201 (35.6%)	84 (35.7%)			
Socioeconomic Status	Low	260 (46.0%)	104 (44.3%)	-	19.69	< 0.001
	Middle	226 (40.0%)	69 (29.4%)			
	High	79 (14.0%)	62 (26.4%)			
Residence	Rural	253 (44.8%)	88 (37.4%)	1.355 (0.992–1.850)	3.65	0.056
	Urban	312 (55.2%)	147 (62.6%)			
BMI (mean±SD) kg/m²		27.12 ± 4.71	25.26 ± 5.05	1.86 (1.13 to 2.60)	4.994	< 0.001
BMI (kg/m²)	<18.5 (Underweight)	22 (3.9%)	13 (5.5%)	-	13.15	0.004
	18.5–24.9 (Normal)	168 (29.7%)	98 (41.7%)			
	25.0–29.9 (Overweight)	213 (37.7%)	72 (30.6%)			
	≥30.0 (Obese)	162 (28.7%)	52 (22.1%)			
Smoking Status	Ever smoker	253 (44.8%)	86 (36.6%)	1.405 (1.027–1.921)	4.55	0.033
	Never smoker	312 (55.2%)	149 (63.4%)			
Time to Hospital Arrival (mean±SD) hours		10.01 ± 4.94	6.52 ± 3.36	3.49 (2.79 to 4.18)	9.906	< 0.001
Arrived ≤4.5 hours	No	501 (88.7%)	170 (72.3%)	2.993 (2.034–4.405)	32.73	< 0.001
	Yes	64 (11.3%)	65 (27.7%)			
Hypertension	No	398 (70.4%)	146 (62.1%)	1.453 (1.055–2.000)	5.27	0.022
	Yes	167 (29.6%)	89 (37.9%)			
Dyslipidemia	No	414 (73.3%)	182 (77.4%)	0.798 (0.558–1.142)	1.52	0.217
	Yes	151 (26.7%)	53 (22.6%)			
Diabetes Mellitus	No	424 (75.0%)	182 (77.4%)	0.876 (0.611–1.256)	0.52	0.470
	Yes	141 (25.0%)	53 (22.6%)			
Chronic Kidney Disease	No	526 (93.1%)	209 (88.9%)	1.678 (0.996–2.826)	3.85	0.050
	Yes	39 (6.9%)	26 (11.1%)			
Lesion Site	Cortical (cerebral cortex)	166 (29.4%)	4 (1.7%)	-	140.4	< 0.001
	Subcortical (white matter, IC)	131 (23.2%)	47 (20.0%)			
	Basal ganglia	134 (23.7%)	67 (28.5%)			
	Thalamus	46 (8.1%)	58 (24.7%)			
	Brainstem	36 (6.4%)	47 (20.0%)			
	Cerebellum	21 (3.7%)	12 (5.1%)			
	Multifocal	31 (5.5%)	0 (0.0%)			
Seizures at Presentation	No	517 (91.5%)	195 (83.0%)	2.209 (1.408–3.468)	12.32	< 0.001
	Yes	48 (8.5%)	40 (17.0%)			
GCS Score on Admission		10.04 ± 2.46	9.27 ± 2.47	0.77 (0.39 to 1.15)	4.025	< 0.001
Systolic Blood Pressure (mmHg)		154.00 ± 31.60	185.30 ± 29.36	-31.30 (-36.02 to -26.58)	-13.023	< 0.001
Diastolic Blood Pressure (mmHg)		98.77 ± 30.83	108.78 ± 31.56	-10.01 (-14.74 to -5.28)	-4.156	< 0.001
Total Cholesterol (mg/dL)		193.94 ± 36.48	180.45 ± 34.70	13.49 (8.01 to 18.97)	4.831	< 0.001
LDL Cholesterol (mg/dL)		126.65 ± 18.16	117.33 ± 24.86	9.32 (6.22 to 12.42)	5.898	< 0.001
HDL Cholesterol (mg/dL)		42.20 ± 4.90	43.43 ± 4.91	-1.23 (-1.97 to -0.48)	-3.224	0.001
Triglycerides (mg/dL)		153.24 ± 35.09	139.11 ± 27.42	14.13 (9.10 to 19.16)	5.513	< 0.001
HbA1c (%)		7.13 ± 2.14	6.95 ± 2.06	0.18 (-0.14 to 0.50)	1.085	0.278
Blood Urea (mg/dL)		49.41 ± 59.86	61.37 ± 86.56	-11.96 (-22.44 to -1.48)	-2.241	0.025
Serum Uric Acid (mg/dL)		7.11 ± 1.22	5.92 ± 1.08	1.18 (1.00 to 1.36)	12.917	< 0.001
Serum Sodium (mEq/L)		136.33 ± 8.68	133.50 ± 9.91	2.82 (1.44 to 4.20)	4.016	< 0.001

In-hospital mortality occurred in 16.4% of patients overall, with a significantly higher rate among hemorrhagic stroke patients (26.0%) compared to ischemic stroke patients (12.4%) (OR=2.48, 95% CI: 1.69-3.64; p<0.001). In-hospital mortality was consistently higher among hemorrhagic stroke patients compared to ischemic stroke across most demographic, socioeconomic, clinical, and radiological categories. Younger age, male gender, lower and middle socioeconomic status, rural or urban residence, overweight or obese BMI, and history of smoking all showed significantly higher odds of mortality with hemorrhagic stroke (OR range: 1.7-6.2, p < 0.05). Clinical comorbidities such as hypertension, diabetes, dyslipidemia, and chronic kidney disease further increased the risk of mortality in hemorrhagic stroke, with seizures at presentation associated with the highest odds (OR = 10.6, 95% CI: 3.27-34.57, p < 0.001). Mortality also varied significantly by lesion site, being particularly elevated in the basal ganglia for hemorrhagic stroke. Early arrival within 4.5 hours and adverse clinical features such as low Glasgow Coma Scale, hypertension, and seizures further amplified mortality risk in the hemorrhagic group, consistently supported by statistically significant odds ratios and confidence intervals (Table 3).

**Table 3:** Analysis of Effect Modifiers and Confounding Variables on In-Hospital Mortality Among Ischemic and Hemorrhagic Stroke Patients (n=800). For each subgroup, the table presents frequencies and column-wise percentages, with odds ratios (OR) and 95% confidence intervals (CI), chi-square (χ²) statistics, and exact p-values illustrating the strength and significance of associations. The reference category is “No” for binary variables and the first subgroup for variables with more than two levels. OR > 1 indicates higher odds of mortality. Statistical significance is defined by a 95% CI excluding 1 and p < 0.05. Abbreviations: BMI, body mass index

Variable (Subgroup)	Stroke Subtype	In-hospital Mortality No (n=669) n (%)	In-hospital Mortality Yes (n=131) n (%)	Odds Ratio (95% CI)	Pearson Chi-square	p-value
Age ≤50 years	Ischemic	95 (92.2%)	8 (7.8%)	4.033 (1.643 to 9.900)	10.226	0.001
	Hemorrhagic	53 (74.6%)	18 (25.4%)			
Age >50 years	Ischemic	400 (86.6%)	62 (13.4%)	2.293 (1.478 to 3.556)	14.204	< 0.001
	Hemorrhagic	121 (73.8%)	43 (26.2%)			
Male	Ischemic	325 (89.3%)	39 (10.7%)	3.106 (1.905 to 5.064)	21.979	< 0.001
	Hemorrhagic	110 (72.8%)	41 (27.2%)			
Female	Ischemic	170 (84.6%)	31 (15.4%)	1.714 (0.911 to 3.222)	2.836	0.092
	Hemorrhagic	64 (76.2%)	20 (23.8%)			
Low socioeconomic status	Ischemic	215 (82.7%)	45 (17.3%)	1.937 (1.138 to 3.298)	6.046	0.014
	Hemorrhagic	74 (71.2%)	30 (28.8%)			
Middle socioeconomic status	Ischemic	208 (92.0%)	18 (8.0%)	6.163 (3.088 to 12.298)	31.136	< 0.001
	Hemorrhagic	45 (65.2%)	24 (34.8%)			
Highsocioeconomic status	Ischemic	72 (91.1%)	7 (8.9%)	1.309 (0.434 to 3.952)	0.229	0.632
	Hemorrhagic	55 (88.7%)	7 (11.3%)			
Rural	Ischemic	223 (88.1%)	30 (11.9%)	2.788 (1.523 to 5.102)	11.640	0.001
	Hemorrhagic	64 (72.7%)	24 (27.3%)			
Urban	Ischemic	272 (87.2%)	40 (12.8%)	2.287 (1.389 to 3.767)	10.915	0.001
	Hemorrhagic	110 (74.8%)	37 (25.2%)			
BMI <18.5 (Underweight)	Ischemic	18 (81.8%)	4 (18.2%)	2.813 (0.593 to 13.336)	1.759	0.185
	Hemorrhagic	8 (61.5%)	5 (38.5%)			
BMI 18.5-24.9 (Normal)	Ischemic	145 (86.3%)	23 (13.7%)	2.045 (1.082 to 3.865)	4.962	0.026
	Hemorrhagic	74 (75.5%)	24 (24.5%)			
BMI 25.0-29.9 (Overweight)	Ischemic	185 (86.9%)	28 (13.1%)	2.641 (1.335 to 5.224)	8.226	0.004
	Hemorrhagic	52 (72.2%)	20 (27.8%)			
BMI ≥30.0 (Obese)	Ischemic	147 (90.7%)	15 (9.3%)	2.940 (1.275 to 6.781)	6.817	0.009
	Hemorrhagic	40 (76.9%)	12 (23.1%)			
Smoking: Ever	Ischemic	221 (87.4%)	32 (12.6%)	2.993 (1.657 to 5.404)	13.994	< 0.001
	Hemorrhagic	60 (69.8%)	26 (30.2%)			
Smoking: Never	Ischemic	274 (87.8%)	38 (12.2%)	2.214 (1.331 to 3.681)	9.679	0.002
	Hemorrhagic	114 (76.5%)	35 (23.5%)			
Non-hypertensive	Ischemic	352 (88.4%)	46 (11.6%)	1.215 (0.692 to 2.133)	0.459	0.498
	Hemorrhagic	126 (86.3%)	20 (13.7%)			
Hypertensive	Ischemic	143 (85.6%)	24 (14.4%)	5.089 (2.792 to 9.279)	30.790	< 0.001
	Hemorrhagic	48 (53.9%)	41 (46.1%)			
Non-diabetic	Ischemic	395 (93.2%)	29 (6.8%)	2.476 (1.426 to 4.300)	10.912	0.001
	Hemorrhagic	154 (84.6%)	28 (15.4%)			
Diabetic	Ischemic	100 (70.9%)	41 (29.1%)	4.024 (2.072 to 7.816)	17.980	< 0.001
	Hemorrhagic	20 (37.7%)	33 (62.3%)			
Non-dyslipidemic	Ischemic	387 (93.5%)	27 (6.5%)	3.293 (1.920 to 5.648)	20.346	< 0.001
	Hemorrhagic	148 (81.3%)	34 (18.7%)			
Dyslipidemic	Ischemic	108 (71.5%)	43 (28.5%)	2.608 (1.370 to 4.967)	8.785	0.003
	Hemorrhagic	26 (49.1%)	27 (50.9%)			
Chronic Kidney Disease: No	Ischemic	473 (89.9%)	53 (10.1%)	2.178 (1.397 to 3.395)	12.208	< 0.001
	Hemorrhagic	168 (80.4%)	41 (19.6%)			
Chronic Kidney Disease: Yes	Ischemic	22 (56.4%)	17 (43.6%)	4.314 (1.421 to 13.094)	7.069	0.008
	Hemorrhagic	6 (23.1%)	20 (76.9%)			
Arrival >4.5h: No	Ischemic	447 (89.2%)	54 (10.8%)	2.069 (1.293 to 3.311)	9.473	0.002
	Hemorrhagic	136 (80.0%)	34 (20.0%)			
Arrival ≤4.5h: Yes	Ischemic	48 (75.0%)	16 (25.0%)	2.132 (1.006 to 4.516)	3.969	0.046
	Hemorrhagic	38 (58.5%)	27 (41.5%)			
Seizures at Presentation: No	Ischemic	469 (90.7%)	48 (9.3%)	1.437 (0.859 to 2.403)	1.924	0.165
	Hemorrhagic	170 (87.2%)	25 (12.8%)			
Seizures at Presentation: Yes	Ischemic	26 (54.2%)	22 (45.8%)	10.636 (3.272 to 34.571)	18.942	< 0.001
	Hemorrhagic	4 (10.0%)	36 (90.0%)			
Lesion Site: Cortical	Ischemic	156 (94.0%)	10 (6.0%)	1.400 (1.005 to 1.950)	45.645	< 0.001
	Hemorrhagic	4 (100.0%)	0 (0.0%)			
Lesion Site: Subcortical	Ischemic	114 (87.0%)	17 (13.0%)	1.588 (0.654 to 3.858)	1.056	0.304
	Hemorrhagic	38 (80.9%)	9 (19.1%)			
Lesion Site: Basal ganglia	Ischemic	116 (86.6%)	18 (13.4%)	2.551 (1.233 to 5.278)	6.625	0.010
	Hemorrhagic	48 (71.6%)	19 (28.4%)			
Lesion Site: Thalamus	Ischemic	43 (93.5%)	3 (6.5%)	1.352 (0.306 to 5.981)	0.159	0.690
	Hemorrhagic	53 (91.4%)	5 (8.6%)			
Lesion Site: Brainstem	Ischemic	16 (44.4%)	20 (55.6%)	0.593 (0.247 to 1.422)	1.380	0.240
	Hemorrhagic	27 (57.4%)	20 (42.6%)			
Lesion Site: Cerebellum	Ischemic	20 (95.2%)	1 (4.8%)	10.000 (0.964 to 103.779)	4.849	0.028
	Hemorrhagic	4 (33.3%)	8 (66.7%)			
Lesion Site: Multifocal	Ischemic	30 (96.8%)	1 (3.2%)	-	-	-
	Hemorrhagic	0 (0.0%)	0 (0.0%)			

In both ischemic and hemorrhagic stroke, lower admission Glasgow Coma Scale (GCS) score and higher systolic blood pressure were strongly associated with increased in-hospital mortality (both p < 0.001). Higher total cholesterol, triglycerides, and HbA1c levels were significant predictors of mortality in both groups (all p < 0.05), as was markedly elevated blood urea (p < 0.001). In ischemic stroke, lower serum sodium was also associated with mortality (p < 0.001), while in hemorrhagic stroke, lower HDL cholesterol (p = 0.006) and higher systolic blood pressure remained significant. No significant differences were found for LDL cholesterol or serum uric acid in either group (Table 4).

**Table 4:** Comparison of Clinical and Laboratory Variables by In-Hospital Mortality Status Among Ischemic and Hemorrhagic Stroke Patients (n=800). Means and standard deviations of baseline clinical and laboratory parameters were compared between in-hospital mortality groups for both stroke types using independent samples t-test. Statistical significance was set at p < 0.05. Negative mean differences reflect higher values among survivors. Abbreviations: CI, confidence interval; SD, standard deviation; BMI, body mass index; GCS, Glasgow Coma Scale; LDL, low-density lipoprotein; HDL, high-density lipoprotein; HbA1c, glycated hemoglobin

Variable	Stroke Subtype	In-hospital Mortality No, Mean ± SD	In-hospital Mortality Yes, Mean ± SD	Mean Difference (95% CI)	t-test	p-value
Age in Years	Ischemic	64.52 ± 11.81	73.91 ± 14.65	-9.40 (-12.46 to -6.34)	-6.034	< 0.001
	Hemorrhagic	59.28 ± 14.33	60.70 ± 14.49	-1.43 (-5.64 to 2.78)	-0.668	0.505
BMI (kg/m²)	Ischemic	27.30 ± 4.64	25.86 ± 5.04	1.44 (0.27 to 2.62)	2.409	0.016
	Hemorrhagic	25.12 ± 5.15	25.64 ± 4.76	-0.52 (-2.00 to 0.96)	-0.689	0.491
Time to Hospital Arrival (hours)	Ischemic	10.04 ± 4.80	9.75 ± 5.86	0.29 (-0.94 to 1.53)	0.467	0.641
	Hemorrhagic	6.72 ± 3.36	5.94 ± 3.32	0.79 (-0.20 to 1.77)	1.575	0.117
GCS Score on Admission	Ischemic	10.25 ± 2.40	8.60 ± 2.44	1.65 (1.04 to 2.25)	5.366	< 0.001
	Hemorrhagic	9.75 ± 2.03	7.92 ± 3.08	1.83 (1.14 to 2.52)	5.242	< 0.001
Systolic Blood Pressure (mmHg)	Ischemic	151.45 ± 29.73	172.00 ± 38.21	-20.55 (-28.29 to -12.80)	-5.208	< 0.001
	Hemorrhagic	180.75 ± 27.96	198.28 ± 29.62	-17.53 (-25.86 to -9.21)	-4.150	< 0.001
Diastolic Blood Pressure (mmHg)	Ischemic	99.35 ± 30.25	94.64 ± 34.61	4.71 (-3.02 to 12.44)	1.197	0.232
	Hemorrhagic	106.49 ± 27.80	115.33 ± 39.94	-8.84 (-18.04 to 0.36)	-1.893	0.060
Total Cholesterol (mg/dL)	Ischemic	192.53 ± 36.22	203.94 ± 37.06	-11.42 (-20.53 to -2.31)	-2.462	0.014
	Hemorrhagic	173.66 ± 27.08	199.82 ± 45.46	-26.16 (-35.78 to -16.54)	-5.358	< 0.001
LDL Cholesterol (mg/dL)	Ischemic	126.79 ± 18.41	125.66 ± 16.35	1.13 (-3.43 to 5.69)	0.486	0.627
	Hemorrhagic	116.67 ± 24.94	119.21 ± 24.75	-2.55 (-9.84 to 4.75)	-0.687	0.492
HDL Cholesterol (mg/dL)	Ischemic	42.32 ± 4.75	41.37 ± 5.86	0.94 (-0.28 to 2.17)	1.509	0.132
	Hemorrhagic	43.94 ± 4.57	41.95 ± 5.53	1.99 (0.57 to 3.41)	2.767	0.006
Triglycerides (mg/dL)	Ischemic	151.60 ± 33.22	164.84 ± 44.78	-13.24 (-21.98 to -4.50)	-2.976	0.003
	Hemorrhagic	136.30 ± 24.67	147.11 ± 33.00	-10.81 (-18.74 to -2.88)	-2.685	0.008
HbA1c (%)	Ischemic	6.91 ± 1.93	8.69 ± 2.83	-1.77 (-2.29 to -1.26)	-6.730	< 0.001
	Hemorrhagic	6.45 ± 1.42	8.40 ± 2.80	-1.96 (-2.51 to -1.41)	-7.022	< 0.001
Blood Urea (mg/dL)	Ischemic	43.69 ± 47.28	89.84 ± 106.72	-46.15 (-60.69 to -31.62)	-6.238	< 0.001
	Hemorrhagic	36.90 ± 28.01	131.18 ± 142.37	-94.28 (-116.61 to -71.95)	-8.319	< 0.001
Serum Uric Acid (mg/dL)	Ischemic	7.12 ± 1.21	7.03 ± 1.28	0.21 (-0.41 to 0.82)	0.602	0.547
	Hemorrhagic	5.97 ± 1.10	5.79 ± 1.01	0.18 (-0.14 to 0.49)	1.101	0.272
Serum Sodium (mEq/L)	Ischemic	136.82 ± 8.28	132.86 ± 10.54	3.96 (1.80 to 6.11)	3.610	< 0.001
	Hemorrhagic	134.06 ± 9.54	131.90 ± 10.84	2.16 (-0.74 to 5.06)	1.469	0.143

## Discussion

The present study found that ischemic stroke constituted 70.6% of all stroke cases, while hemorrhagic stroke accounted for 29.4%, a distribution closely consistent with previous international and regional epidemiological reports [1,4,6]. Globally, ischemic stroke remains the predominant subtype, accounting for 62-88% of all incident strokes, while hemorrhagic stroke comprises 12-38% of cases [12]. European and Middle Eastern cohorts generally report ischemic stroke frequencies of 80-85% and hemorrhagic stroke at 15-20% [13,14]. In Asia, and specifically in East and Southeast Asia, the proportion of hemorrhagic stroke is somewhat higher, yet ischemic stroke still predominates, with large Chinese and South Asian studies indicating ischemic stroke rates around 79-88% and hemorrhagic stroke 12-21% [15,16]. The higher frequency of hemorrhagic and overall stroke cases at this tertiary care hospital likely results from its status as the largest neuroscience referral center, where patients from multiple regions with more severe or complex presentations are concentrated, leading to elevated observed rates compared to non-referral or general hospital settings.

Patients with hemorrhagic stroke were observed to be significantly younger than those with ischemic stroke, consistent with previous reports indicating mean age differences of seven to 10 years between the subtypes [17,18]. This age disparity has been documented across multiple populations and is attributed to differing risk factor profiles, with hypertension and diabetes more frequently observed in hemorrhagic stroke patients, while ischemic stroke is more often linked to atrial fibrillation. Regarding gender, men comprised a higher proportion of both ischemic and hemorrhagic stroke cases, particularly intracerebral hemorrhage [19,20]. Ischemic stroke predominated in both rural and urban populations, with a higher ischemic-to-hemorrhagic ratio in rural (4.3:1) than urban (3.8:1) areas, matching prior registry data [15]. Lower socioeconomic status was significantly linked to increased ischemic stroke risk, poorer outcomes, and higher long-term mortality, whereas this association was weaker for hemorrhagic stroke, consistent with published evidence [21,22]. This disparity may be attributed to differential access to preventive healthcare, delayed presentation, and lower adherence to long-term secondary prevention strategies among lower socioeconomic groups, particularly impacting ischemic stroke outcomes.

Patients with ischemic stroke in this study exhibited significantly higher BMI than those with hemorrhagic stroke, in agreement with prior reports identifying overweight and obesity as key risk factors for ischemic events. Rising BMI correlates with increased ischemic stroke risk, while a U-shaped pattern links both low and high BMI to higher hemorrhagic stroke risk in men, but less so in women. Class 1 obesity is specifically linked to ischemic, not hemorrhagic stroke [9,23].

Hypertension was significantly more frequent among hemorrhagic stroke patients in this study, with an odds ratio of 1.45 (95% CI: 1.05-2.00, p = 0.022), consistent with previous literature describing hypertension rates nearing 100% in hemorrhagic stroke patients and slightly less prevalent in ischemic stroke (60-90%) [6,7]. Diabetes mellitus and dyslipidemia, by contrast, showed higher prevalence among ischemic stroke patients, as supported by studies reporting diabetes in 30-50% of ischemic cases versus 10-30% in hemorrhagic, and dyslipidemia in 35-80% of ischemic versus 10-40% of hemorrhagic strokes [5,6]. While the present study did not observe significant differences in frequency for diabetes or dyslipidemia between groups, ischemic stroke patients exhibited higher mean total and low-density lipoprotein cholesterol, in line with global data highlighting lipid abnormalities as more prominent in ischemic stroke and potentially inversely associated with hemorrhagic stroke [5-7]. Chronic kidney disease was found in 8.1% of cases, and its distribution by stroke subtype indicated that ischemic stroke is more common in chronic kidney disease [24].

Hemorrhagic stroke patients in this study presented to the hospital significantly earlier than ischemic stroke patients, mirroring registry data that report a median arrival time of 248 minutes for hemorrhagic stroke versus 483 minutes for ischemic stroke, and a 42% rate of early (≤4.5 hours) arrival for hemorrhagic cases compared to 30% in ischemic stroke [7,18]. Early presentation is especially crucial for timely intervention in ischemic stroke, where treatment efficacy is time-dependent. Lesion analysis revealed ischemic stroke was most frequently localized to the basal ganglia (25.1%), subcortical (22.3%), and cortical (21.3%) regions, whereas hemorrhagic stroke more often affected deep structures such as basal ganglia, thalamus, brainstem, and cerebellum, corroborating previous findings that deep brain involvement predominates in hemorrhagic events [10,25]. Regarding neurological status, hemorrhagic stroke was associated with lower mean GCS scores at admission (9.27 ± 2.47) compared to ischemic stroke (10.04 ± 2.46), supporting previous observations of greater initial severity and higher mortality in hemorrhagic stroke [25,26]. Admission blood pressure was also significantly higher in the hemorrhagic group, with systolic averaging 185.3 mmHg and diastolic 108.8 mmHg, consistent with published data [27].

A previous study reported that serum uric acid level consistently found higher in ischemic stroke compared to hemorrhagic stroke, reporting mean of 303 μmol/L in ischemic versus 269 μmol/L in hemorrhagic stroke [28]. For serum sodium, findings are heterogeneous; several studies indicate lower sodium levels and less frequent hyponatremia in ischemic stroke, whereas hyponatremia is more common and sodium values higher in hemorrhagic stroke, with some studies reporting a significant difference (129.4 ± 3.1 mEq/L in ischemic versus 134.4 ± 3.5 mEq/L in hemorrhagic, p < 0.0001) [29,30]. These metabolic disturbances have important prognostic implications, particularly for acute management and outcome prediction.

Consistent with previous large multicenter and registry-based studies, the present study demonstrated a significantly higher in-hospital mortality rate for hemorrhagic stroke (26.0%) compared to ischemic stroke (12.4%), with the odds of mortality being more than twice as high in hemorrhagic cases (OR=2.48, 95% CI: 1.69-3.64; p<0.001). In-hospital mortality for ischemic stroke typically ranges from 3.5% to 6.7%, whereas mortality for hemorrhagic stroke spans from 15.1% to 22.5% and can reach up to 39% in critically ill populations [10,18,24,25]. Early mortality is particularly pronounced in hemorrhagic stroke, with the risk of death within the first week being nearly four times greater than that of ischemic stroke [23,25]. Lower Glasgow Coma Scale scores, especially in the context of hemorrhagic stroke and deep brain involvement, are powerful predictors of mortality [5,10,25]. Although overall lesion-specific mortality is higher in hemorrhagic stroke, current literature lacks detailed site-by-site mortality data for each subtype, underscoring the importance of further research in this area.

This study's major strengths include its large sample size and the detailed assessment of key demographic, metabolic, and neurological variables. The consecutive enrollment and broad inclusion of acute stroke presentations from a high-volume tertiary care center enhance generalizability to similar referral settings. However, several limitations must be acknowledged. The cross-sectional design restricts the ability to establish temporal or causal relationships between risk factors and stroke subtypes. Moreover, the study was conducted at a single tertiary care center in Pakistan, which may limit ethnic and regional generalizability. As such, the findings may not fully represent the broader diversity of stroke presentations across different ethnic and socioeconomic groups. Future studies employing multicenter, prospective designs and incorporating long-term outcomes are needed to clarify causal pathways, identify modifiable risk factors, and optimize acute and post-stroke care strategies tailored to regionally diverse patient populations.

## Conclusions

This study demonstrates that ischemic and hemorrhagic strokes have distinctly different clinical and metabolic profiles among adults presenting to a tertiary care center. Ischemic stroke was more frequently observed, particularly in older adults and those with a history of smoking, while hemorrhagic stroke was associated with higher blood pressure, greater representation among individuals of higher socioeconomic status, and more rapid hospital arrival after symptom onset. The findings also highlight that hypertension remains a key risk factor for hemorrhagic stroke, whereas elevated cholesterol and triglyceride levels are more closely related to ischemic stroke. Importantly, in-hospital mortality was substantially higher in the hemorrhagic stroke group, with mortality risk amplified by advanced age, reduced consciousness at admission, and unfavorable metabolic findings. These insights emphasize the need for risk factor modification and tailored acute management strategies, with particular focus on blood pressure control and early intervention, to reduce adverse outcomes in both stroke subtypes within the local population.
